# Correction to: Linking meta-omics to the kinetics of denitrification intermediates reveals pH-dependent causes of N_2_O emissions and nitrite accumulation in soil

**DOI:** 10.1038/s41396-021-01100-y

**Published:** 2021-12-17

**Authors:** Åsa Frostegård, Silas H. W. Vick, Natalie Y. N. Lim, Lars R. Bakken, James P. Shapleigh

**Affiliations:** 1 Faculty of Chemistry, Biotechnology and Food Science, Norwegian University of Life Sciences, Ås, Norway; 2 Department of Microbiology, Cornell University, Ithaca, NY USA

**Keywords:** Metagenomics, Soil microbiology

Correction to: *The ISME Journal* 10.1038/s41396-021-01045-2, published online 1 July 2021

Following the publication of this article, the authors noted an error in Table 1. In the first column it should be DNA, not RNA.

The original article has been corrected.

