# Supplementary material for: Linking meta-omics to the kinetics of denitrification intermediates reveals pH-dependent causes of N2O emissions and nitrite accumulation in soil
Source: ISME J. 2021 Jul 1;16(1):26–37. doi: 10.1038/s41396-021-01045-2 (PMC8692524; doi:10.1038/s41396-021-01045-2)
Supplement: Supplementary file 1 — Figures S1, S2 and HONO calculations [file 41396_2021_1045_MOESM1_ESM.pdf]

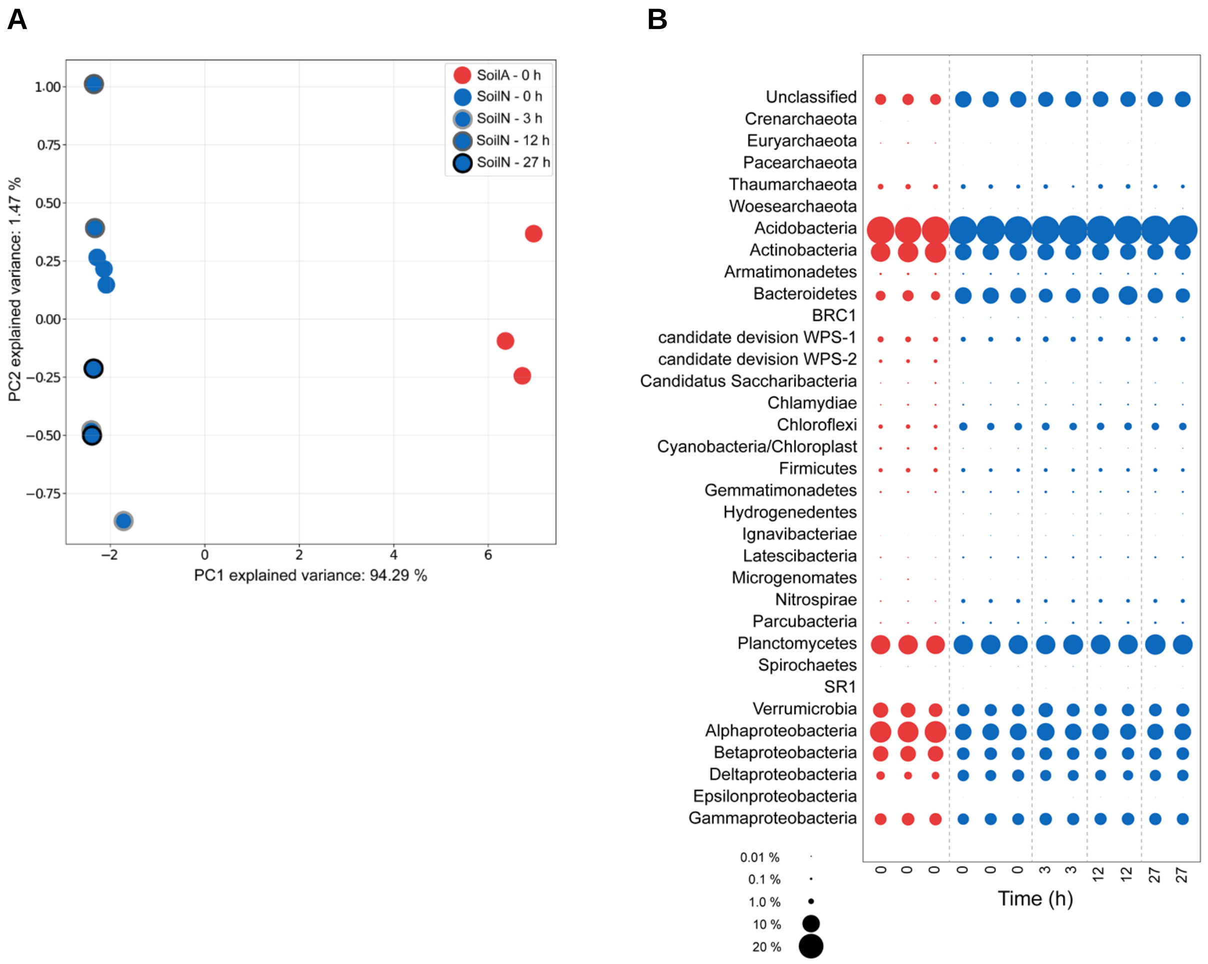

Fig. S1. Taxonomic distribution of bacteria in SoilA and SoilN based on 16S rRNA gene sequences. A Principal component analysis (PCA) based on the relative abundance of OTUs. **B** Taxonomic breakdown. Initial time point samples (0 h incubation, both soils; n=3). For SoilN these were compared against grouped samples taken after 3, 12 and 27 hours of incubation (n=2). Samples were sequenced using MiSeq technology (Illumina) after amplification of the V4 region of the 16S rRNA gene (primers 515f/806R).

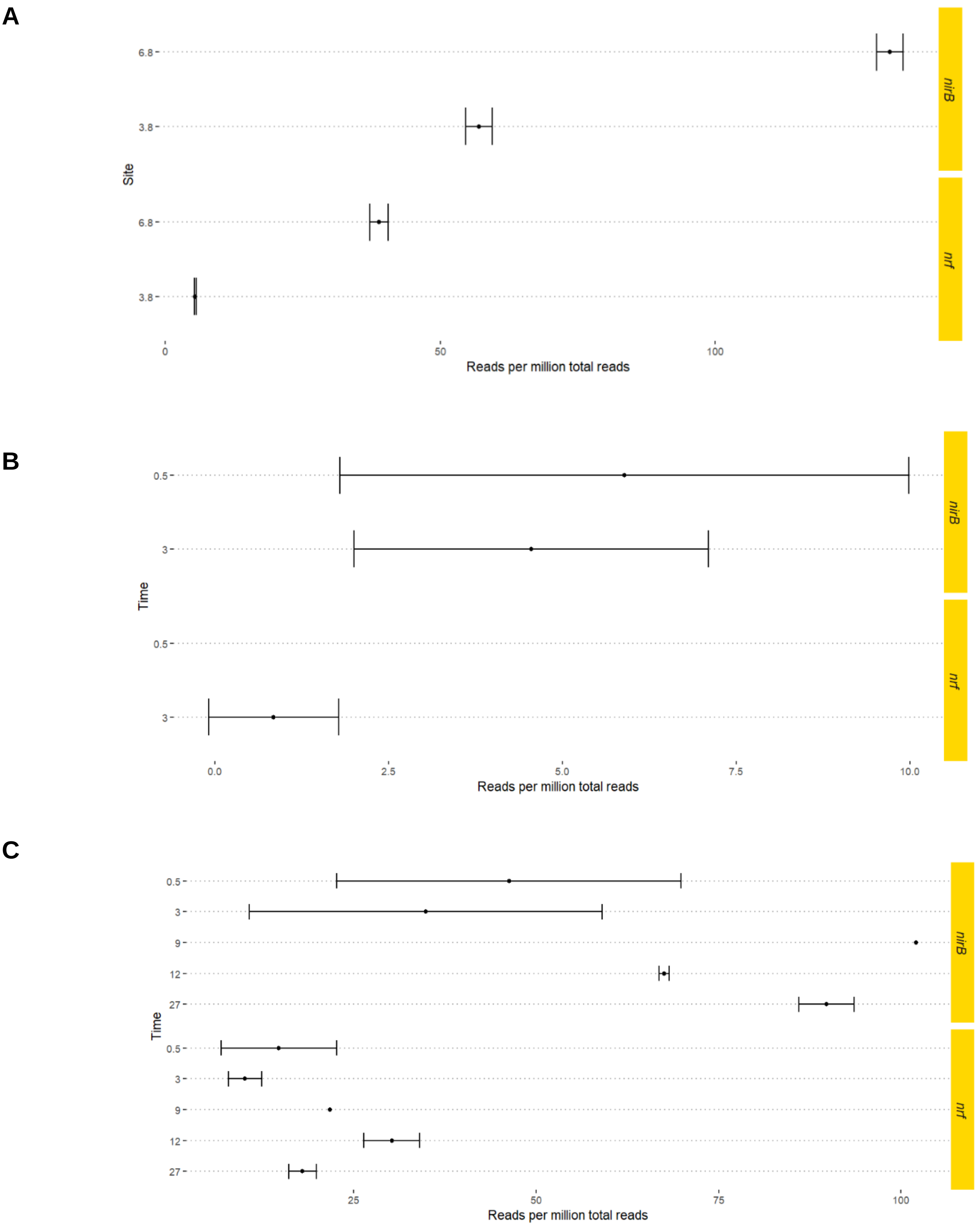

Fig. S2. Genetic potential and transcription of the DNRA-related genes *nrfA* and *nirB* in the metagenome and metatranscriptome. A Average gene read abundances (as reads per million, RPM). Bars show sd (n=3). **B** Average transcript read abundances after 0.5 and 3h (SoilA). **C** Average transcript read abundances after 0.5, 3, 9, 12 and 27 h of incubation (SoilN). **B** and **C** (transcript reads): Duplicate samples were analyzed for each sampling point, bars show highest and lowest value.

Calculating the concentration of HNO_2 as a function of pH and total nitrite concentration.

The Henderson-Hasselbalch equation (1) can be used to calculate the relative amount of undissociated nitrite as a function of pH (which is controlled by the buffer system of the soil):

$K_a = [H^+] * [A^-]/[HA]$ (1)

where HA is an acid that dissociates into A^- and H^+ and K_a is the acid dissociation constant.

Taking the $\log_{10}$ of both sides
$\log_{10}(K_a) = \log_{10}[H^+] + \log_{10}([A^-]/[HA])$ (2)

defining $pX = -\log_{10}[X]$, (2) gives:

$-pK_a = -pH + \log_{10}([A^-]/[HA])$ (3)

Replacing $[A^-]$ with $[NO_2^-]$ and [HA] with $[HNO_2]$,
and solving equation (3) for $[NO_2^-]/[HNO_2]$:

$[NO_2^-]/[HNO_2] = 10^{(pH-pKa)}$ (4)

Equation (4) can be solved for $[HNO_2]/([HNO_2] + [NO_2^-])$, which is the molar fraction of total nitrite (as measured) that is undissociated:

$[HNO_2]/([HNO_2] + [NO_2^-]) = 1/(1+[NO_{2-}]) = 1/(1+10^{(pH - pKa)})$ (5)

Hence, we can calculate the concentration of undissociated HNO_2 in the soil
$[HNO_2] = TNN/(1+10^{(pH - pKa)})$ (6)

where TNN is the measured concentration of total nitrite N ($[HNO_2] + [NO_2^-]$, pH is the measured soil pH and K_a is the dissociation constant for nitrous acid, which is 4E-4 (hence pK_a=3.3398).

Needless to say, soil pH is the most problematic parameter, since the pH may vary throughout the soil matrix, and the bulk pH as measured depends on the cation concentration in the soil slurry. Our pH measurements were done in 0.01 M $CaCl_2$, which is thought to give pH values close to the average of the intact soil. Higher salt concentrations will give lower pH values and *vice versa*.
