# Supplementary material for: Linking meta-omics to the kinetics of denitrification intermediates reveals pH-dependent causes of N2O emissions and nitrite accumulation in soil
Source: ISME J. 2021 Jul 1;16(1):26–37. doi: 10.1038/s41396-021-01045-2 (PMC8692524; doi:10.1038/s41396-021-01045-2)

**Table S2.** **Denitrification gene and transcript prevalence based on data from metagenomics (MG) and metatranscriptomics (MT).** Annotated genes (MG; DNA sampled at start of incubation) and transcripts (MT) sampled after 0.5 and 3 h anoxic incubation (SoilA; soil pH 3.8) and 0.5-27 h anoxic incubation (SoilN; soil pH 6.8). Empty cell: 0 reads.

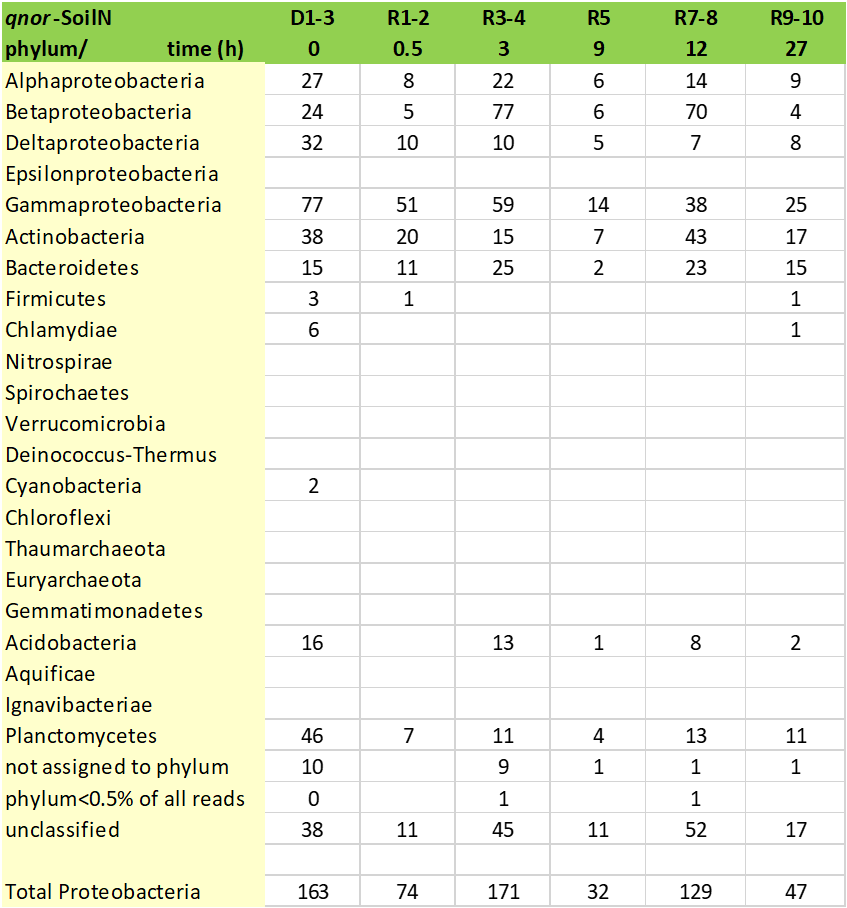

Supplement: Supplementary file 3 — Table S2 [file 41396_2021_1045_MOESM3_ESM.docx]
